# “Open Sesame?”: Biomarker Status of the Human Equilibrative Nucleoside Transporter-1 and Molecular Mechanisms Influencing its Expression and Activity in the Uptake and Cytotoxicity of Gemcitabine in Pancreatic Cancer

**DOI:** 10.3390/cancers12113206

**Published:** 2020-10-31

**Authors:** Ornella Randazzo, Filippo Papini, Giulia Mantini, Alessandro Gregori, Barbara Parrino, Daniel S. K. Liu, Stella Cascioferro, Daniela Carbone, Godefridus J. Peters, Adam E. Frampton, Ingrid Garajova, Elisa Giovannetti

**Affiliations:** 1 Department of Medical Oncology, Cancer Center Amsterdam, Amsterdam UMC, VU University Medical Center (VUmc), 1081 HV Amsterdam, The Netherlands; o.randazzo@amsterdamumc.nl (O.R.); f.papini@student.vu.nl (F.P.); g.mantini@amsterdamumc.nl (G.M.); a.gregori@amsterdamumc.nl (A.G.); gj.peters@amsterdamumc.nl (G.J.P.); igarajova@ao.pr.it (I.G.); 2 Dipartimento di Scienze e Tecnologie Biologiche Chimiche e Farmaceutiche (STEBICEF), Università degli Studi di Palermo, 90123 Palermo, Italy; barbara.parrino@unipa.it (B.P.); stellamaria.cascioferro@unipa.it (S.C.); daniela.carbone@unipa.it (D.C.); 3 Cancer Pharmacology Lab, AIRC Start Up Unit, Fondazione Pisana per la Scienza, 56017 Pisa, Italy; 4 Division of Cancer, Department of Surgery & Cancer, Imperial College, Hammersmith Hospital campus, London W12 0NN, UK; daniel.liu08@imperial.ac.uk; 5 Department of Biochemistry, Medical University of Gdansk, 80-210 Gdansk, Poland; 6 Faculty of Health and Medical Sciences, The Leggett Building, University of Surrey, Guildford GU2 7XH, UK; 7 Medical Oncology Unit, University Hospital of Parma, Via Gramsci 14, 43126 Parma, Italy

**Keywords:** pancreatic cancer, drug resistance, human equilibrative nucleoside transporter 1, clinical outcome

## Abstract

**Simple Summary:**

Despite the enormous advance in biomarker discovery, many potential biomarkers of drug activity are unable to satisfy the clinical need due to inadequate sensitivity and specificity. The nucleoside transporter hENT-1 has been studied as a potential biomarker to predict the effect of the widely used anticancer drug gemcitabine in pancreatic cancer. However, several studies showed controversial results regarding the predictive value of hENT-1, prompting new analyses with larger cohorts of patients and standardized methodologies. Improved insights on molecular mechanisms underlying hENT-1 expression and activity should also help in the identification of subsets of patients who are more likely to benefit from specific treatments and improve their clinical outcome. The establishment of such biomarker is especially valuable in pancreatic cancer, which is frequently characterized by complex disease biology and high mortality.

**Abstract:**

Pancreatic ductal adenocarcinoma (PDAC) is an extremely aggressive tumor characterized by early invasiveness, rapid progression and resistance to treatment. For more than twenty years, gemcitabine has been the main therapy for PDAC both in the palliative and adjuvant setting. After the introduction of FOLFIRINOX as an upfront treatment for metastatic disease, gemcitabine is still commonly used in combination with nab-paclitaxel as an alternative first-line regimen, as well as a monotherapy in elderly patients unfit for combination chemotherapy. As a hydrophilic nucleoside analogue, gemcitabine requires nucleoside transporters to permeate the plasma membrane, and a major role in the uptake of this drug is played by human equilibrative nucleoside transporter 1 (hENT-1). Several studies have proposed hENT-1 as a biomarker for gemcitabine efficacy in PDAC. A recent comprehensive multimodal analysis of hENT-1 status evaluated its predictive role by both immunohistochemistry (with five different antibodies), and quantitative-PCR, supporting the use of the 10D7G2 antibody. High hENT-1 levels observed with this antibody were associated with prolonged disease-free status and overall-survival in patients receiving gemcitabine adjuvant chemotherapy. This commentary aims to critically discuss this analysis and lists molecular factors influencing hENT-1 expression. Improved knowledge on these factors should help the identification of subgroups of patients who may benefit from specific therapies and overcome the limitations of traditional biomarker studies.

## 1. Introduction

In the story of “Ali Baba and the Forty Thieves” from the book *One Thousand and One Nights*, “Open sesame” is the magical phrase that opens the mouth of a cave in which the thieves have hidden a treasure. This statement has been commonly used to define something that allows a person to do or enter something easily, or something that unfailingly brings about a desired end. Several cellular transporters are essential for the entry (or efflux) of anticancer drugs [1,2] but, despite a number of preclinical and clinical studies on their role as biomarkers and targets, they have not yet been exploited or exploited correctly to improve clinical outcome.

The human equilibrative nucleoside transporter (hENT-1) represents a quintessential example of such a transporter. This protein is indeed the main transporter involved in the entrance of nucleoside analogs and has attracted extensive attention for its potential role as predictive biomarker for the anticancer activity of gemcitabine [3], as well as for the development of drugs bypassing this transporter in order to overcome gemcitabine resistance in pancreatic ductal adenocarcinoma (PDAC) [4,5,6]. In particular, we have read with great interest the recent comprehensive multimodal analysis of hENT-1 status, which has been performed by Raffenne and collaborators in the largest cohort of PDAC patients to date [3]. In the present commentary, we summarize the key findings of this analysis and discuss further insights on molecular and pharmacological factors influencing the role of nucleoside transporters in the uptake and cytotoxicity of gemcitabine in PDAC.

## 2. Pancreatic Ductal Adenocarcinoma (PDAC)

Pancreatic ductal adenocarcinoma (PDAC) is the most common form of pancreatic cancer and is amongst the deadliest solid malignancies [7]. Despite extensive genetic mapping elucidating key mechanisms in PDAC initiation and progression [8], both conventional and experimental drugs showed limited effects [7].

PDAC is indeed a highly invasive and aggressive disease with an overall 5-year survival rate lower than 10% [7]. Several factors are responsible for this grim prognosis, including late diagnosis and lack of effective therapies [9,10]. Surgery represents the only curative intervention available for patients with local disease. However, even after successful tumor resection and adjuvant chemotherapy, the 5-year survival rate is around 25–30%, with most of the patients experiencing tumor recurrence and metastatic disease within 6–24 months from surgery [11,12]. Chemotherapy is the only treatment available for patients with advanced or metastatic disease, which includes the vast majority of diagnosed PDAC cases [7].

Unfortunately, PDAC is an inherently resistant disease, with low percentages of response rate to all current treatment regimens. Moreover, even when chemotherapy is initially effective, chemoresistance typically occurs after a few cycles, leading to disease progression and mortality [13,14]. This resistance is caused by both cellular intrinsic and extrinsic factors such as cancer stem cells (CSCs), activation of the epithelial-mesenchymal transition (EMT), and presence of a highly desmoplastic and immunosuppressive tumor microenvironment (TME) [15,16,17,18]. Understanding these molecular mechanisms of chemoresistance is an essential step towards increasing the efficacy of treatments and clinical outcome.

Despite the development of more effective multi-drug regimens such as FOLFIRINOX (a chemotherapy regimen made up of the following four drugs: FOL—folinic acid, F—fluorouracil (5-FU), IRIN—irinotecan, OX—oxaliplatin), gemcitabine is still widely used for PDAC treatment, both combined with nab-paclitaxel (Abraxane^®^, Celgene, Summit, NJ, USA), and as monotherapy, especially in patients who are unfit for more toxic poly-chemotherapy regimens [14]. Several studies have focused on molecular intracellular determinants of gemcitabine activity and metabolism. Among these molecular determinants, mRNA and protein expression of the equilibrative transporter-1 (hENT-1) emerged as potential predictors of drug activity in a number of preclinical and clinical studies in PDAC.

## 3. Nucleoside Transporters Involved in Gemcitabine Uptake

The two major classes of nucleoside transporters that have been described in mammalian cells include the concentrative nucleoside transporters (CNTs) and the equilibrative nucleoside transporters (ENTs). These transporters are transmembrane glycoproteins that localize to the cellular and mitochondrial membranes, but can also be found in lysosomes [19] and mediate the cellular uptake of nucleosides required for nucleotide synthesis in cells that lack de novo nucleotide synthesis pathways.

CNTs mediate the inward Na^+^-dependent transport whereas ENTs are bi-directional facilitators of the transmembrane flux of nucleosides [20]. ENTs can be found in almost all cell types unlike CNTs, which are present in intestinal and renal epithelia [21], as well as in hepatocyte cells and in chorionic villi of human term placenta [22].

As bi-directional carriers, ENTs regulate both the influx and efflux of substrates. The human ENT homologues (hENTs) are classified into four groups: hENT-1 (SLC29A1), hENT-2 (SLC29A2), hENT-3 (SLC29A3) and hENT-4 (SLC29A4) [23]. The hENT-1 is sensitive to nitrobenzylmercaptopurine ribonucleoside (NBMPR) to which it binds with a high affinity [24]; while hENT-2 is insensitive to inhibition by NBMPR. However, the hENT1–3 shows selectivity to the NBMPR substrate which also blocks hENT-4 albeit at higher concentration than required for hENT-1 [25]. Of note, hENT-4 is better known as the plasma membrane monoamine transporter because it carries organic cations such as biogenic amines and neurotoxins. This transporter mediates the transport of adenosine in a pH-dependent manner and its activity increases in acidic conditions (optimal transport at pH 6.0) [26].

**Figure 1:**
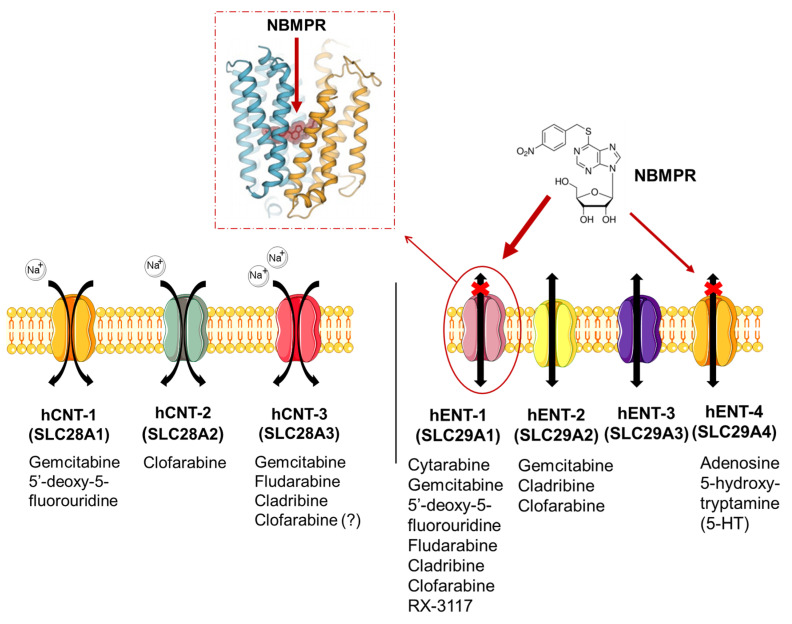
Nucleoside transporters in mammalian cells. CNTs facilitate the Na^+^-dependent transport while ENTs are bi-directional and Na^+^-independent transporters, facilitating the uptake of different anticancer drugs [27,28,29,30]. Of note, hENT-1 (es) is NBMPR-sensitive and the figure shows its crystalized structure [30]. Acronyms: 5-HT, 5-hydroxy-tryptamine; NBMPR, nitrobenzylmercaptopurine ribonucleoside.

These transporters are involved in the uptake of several drugs (Figure 1) with different chemical structures (Figure S1).

Gemcitabine (2′,2′-difluorodeoxycytidine, dFdC) is a pyrimidine analog and relies on membrane transporters for its intracellular uptake [31,32]. The uptake of gemcitabine can be mediated by hENT-1/2 and hCNT-1/3. However, hENT-1 and hCNT-1 appear to be the most efficient transporters involved in the entry of gemcitabine into cells. Notably, hENT-1, the most widely expressed nucleoside transporter in human tissues, is overexpressed in different tumor types, including PDAC (Figure S2A). This is reported in the GEPIA web server, analyzing the RNA sequencing expression data of 9736 tumors and 8587 normal samples from the TCGA and the GTEx projects [33]. However, the comparison of TCGA-PAAD data of pancreatic cancer specimens with the matched non-tumor samples as well as the analysis of similarly matched transcriptomics and proteomics public datasets did not show a significance difference in hENT-1 expression levels (Figure S2B).

Structurally, hENT-1 is a 456-residue protein (50 kDa) with 11 transmembrane domains and three N-linked glycosylation sites, which are essential for localization, function and oligomerization. The first glycosylation site (Asn 48) is located between the first and second transmembrane domains in the hydrophilic loop, whereas the other two sites (Asn 277 and 288) are between the sixth and seventh transmembrane domains [32]. The Km for purine and pyrimidine nucleosides transport range from 0.05 mM to 0.60 mM according to a study performed in *Xenopus laevis* [34].

## 4. Role of hENT-1 in Gemcitabine Activity as Potential Predictive Biomarker

Several studies showed that hENT-1 expression is essential for gemcitabine cytotoxic effects [35,36]. Higher expression levels of hENT-1 have indeed been associated with higher uptake and activity of gemcitabine in cancer cells, using different preclinical models [37,38,39,40].

**Figure 2:**
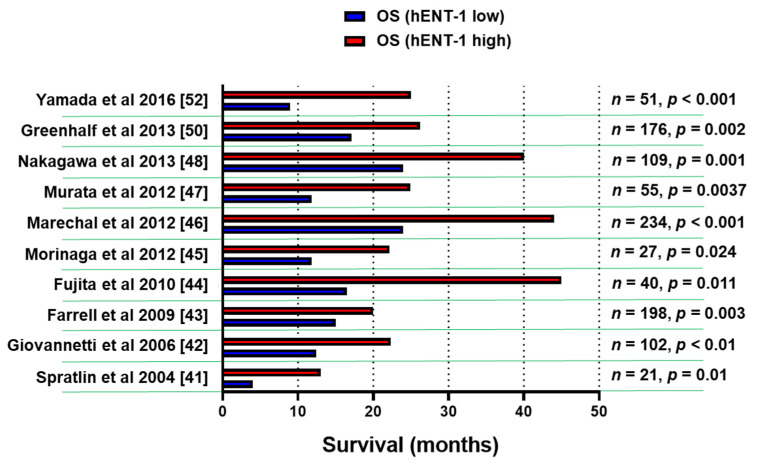
Association of hENT-1 expression levels and survival. Bar graph summarizing the survival data of PDAC patients in studies supporting the role of hENT-1 as a predictive biomarker for the efficacy of gemcitabine. The clinicopathological and treatment details of studies before 2014 [41,42,43,44,45,46,47,48] were reviewed systematically by Nordh et al. [49], while a more recent systematic review by Bird et al. [51] evaluated also the data from the European Study Group for Pancreatic Cancer 3 (ESPAC-3) trial [50]. *n* = number of patients, *p* = statistical *p* values.

A number of retrospective studies on hENT-1 mRNA and protein expression with PCR and immunohistochemical (IHC) methodology demonstrated that high levels of hENT-1 correlated with a statistically significant longer survival (Figure 2), both in the adjuvant and in the metastatic setting, though the number of patients in the latter cohort was extremely small [41,42,43,44,45,46,47,48,49,50,51,52]. For instance, a retrospective analysis of a cohort of PDAC patients from the RTOG9704 phase III clinical trial, which compared gemcitabine with 5-FU after surgical resection, showed an association between high tumor hENT-1 expression and increased overall survival (OS) in patients who received gemcitabine (*n* = 91), but not in those who received 5-FU [43]. These data support the role of hENT-1 as a specific predictive biomarker for the efficacy of gemcitabine.

Conversely, high expression levels of hENT-1 emerged as a prognostic biomarker of poor outcome in cholangiocarcinoma. Indeed, while a first study showed a significant association between disease-free survival (DFS) and high expression of membrane hENT-1 in gemcitabine-treated patients [53,54], hENT-1 overexpression was associated to high proliferation rate and significantly shorter survival in resected intrahepatic cholangiocarcinoma patients who did not receive adjuvant treatments [55]. This might be explained by the different levels of hENT-1 expression and proliferative rates in different tumor types and warrants further, larger studies.

Up to now, the largest prospective study on hENT-1 in PDAC has been performed within the European Study Group for Pancreatic Cancer 3 (ESPAC-3) trial. This study highlighted a significant association between high hENT-1 protein expression and longer OS and DFS in PDAC patients receiving gemcitabine-based chemotherapy post-surgery [50]. Moreover, a quantitative metanalysis including 7 studies with a total of 770 patients (405 hENT-1-negative and 365 hENT-1-positive). showed that hENT-1 expression was significantly associated with both prolonged DFS (HR 0.58, 95% CI, 0.42–0.79) and OS (HR 0.52, 95% CI, 0.38–0.72) in patients receiving adjuvant gemcitabine-based therapy [52].

In contrast, Kawada et al. [56], who evaluated hENT-1 expression in PDAC patients undergoing neoadjuvant chemoradiation, showed only a trend towards statistically significant better disease-specific survival in patients with low expression of hENT-1. These results might be explained by the potential preferential eradication of tumor cells with high expression of hENT-1 by the neoadjuvant treatment before the tumor samples collection. This issue can be overcome by the use of fine-needle aspiration biopsy (FNAB) for retrieving cancer cells before treatment and resection of the tumor. Indeed, a study on pretreatment hENT-1 expression in endoscopic ultrasonography-guided FNAB specimens obtained from resectable, borderline-resectable, and locally advanced PDAC, showed that hENT-1 expression was an independent prognostic factor in both whole patients and those with resection [52]. Regardless of T3 and T4, hENT-1-positive patients with resection had significantly better prognosis than hENT-1-negative patients, whose prognosis was similar to those without resection, suggesting that the evaluation of hENT-1 expression using FNAB samples before chemoradiation provides useful information on patients who might benefit from curative-intent resection.

However, other recent studies on the evaluation of hENT-1 status using IHC in PDAC patients reported conflicting results, possibly due to the use of different antibodies. In particular, the analysis of samples from 156 patients enrolled in the CONKO-001 phase III trial did not show a significant association of high hENT-1 expression with improved median DFS or OS [57]. Similar negative results were observed within a retrospective translational subgroup analysis for hENT-1 in 130 samples from patients enrolled in the AIO-PK0104 multicenter phase III trial [58]. In both cases the researchers used the rabbit monoclonal antibody SP120 and suggested to perform a parallel study using the rabbit 10D7GD antibody. Such study was performed by Marechal and collaborators as well as by Svrcek and collaborators who reported that hENT-1 status was predictive of gemcitabine benefit in patients receiving gemcitabine-adjuvant chemotherapy when evaluated with the 10D7G2 antibody, yet no predictive value was observed when hENT-1 status was assessed using the SP120 antibody [46,59].

In contrast, Kalloger and collaborators, who performed the staining with 10D7G2 and SP120 antibodies (both optimized to run on the Ventana platform), in samples from 227 patients, suggested that both these antibodies can be used to predict gemcitabine sensitivity in resected PDAC [60]. This study suggested also that the use of both antibodies and of the percentage of cells staining positive for hENT-1, instead of the H-score methodology, add critical information that enables the stratification of patients with good, intermediate, or poor response to adjuvant gemcitabine. However, as recognized by the authors: “these findings need to be externally validated in cohorts derived from randomized controlled trials” [60].

Overall, these controversial findings question the predictive value of the available anti-hENT-1 antibodies and call for the establishment of a standardized IHC methodology before hENT-1 status could be used as a predictive biomarker in the clinical setting.

## 5. Evaluation of the Study “hENT-1 Status in PDAC Patients—Are We Ready Yet?”

In a recent study Raffenne and collaborators provided the most comprehensive multimodal analysis of hENT-1 status in the largest cohort of PDAC patients to date (i.e., 471 patients with resected PDAC) [3]. In this study the expression of hENT-1 evaluated using the 10D7G2 antibody was predictive of both prolonged DFS and OS in PDAC patients receiving gemcitabine adjuvant chemotherapy. In contrast, no predictive value of gemcitabine benefit was observed when hENT-1 status was assessed using the SP120 clone when comparing surgery-gemcitabine vs. surgery-only groups. Three additional antibodies (PAB2255, MC-9777, and 11337-1-AP) manufactured by three different companies (MBL™, Woburn, MA, USA; Abnova™, Taipei, Taiwan; and Acris™-OriGene™, Rockville, MD, USA) were further evaluated to establish their potential for the analysis of hENT-1 status. None of these antibodies showed a predictive value of gemcitabine benefit over controls, providing compelling evidence that commercially available anti-hENT-1 antibodies are not suitable for the evaluation of hENT-1 status. Interestingly, all tested antibodies, except the 10D7G2 clone, detected multiple bands on Western blot that did not correspond to the expected glycosylated forms of hENT-1, hence suggesting that these antibodies may recognize and bind to non-completely functional forms of the hENT-1 protein.

Raffenne and collaborators also evaluated the predictive value of mRNA expression levels of hENT-1 which was assessed using microarray data and qRT-PCR analyses performed on formalin-fixed paraffin-embedded (FFPE) tissues from resected specimens. No difference in both DFS and OS was observed when the median hENT-1 mRNA value was used to discriminate between hENT-1 high- and low-expressing tumors. Nonetheless, an increasingly predictive trend was detected when more stringent thresholds were employed (top 25% for OS and top 10% for DFS). Further increase of the threshold (top 10% vs. bottom 10% or bottom 90%) allowed the selection of a population of exceptional gemcitabine responders.

These results might be explained by the fact that the authors used whole tumors. Because of the dense stromal reaction, the analysis of PCR data in PDAC specimens should indeed be performed only after careful evaluation of the percentage of tumor cells and, when feasible, after laser-microdissection [61].

**Figure 3:**
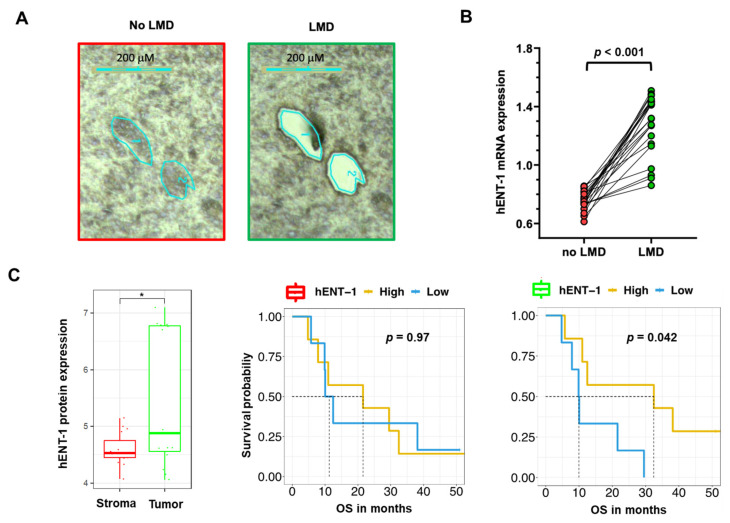
Impact of laser-microdissection on mRNA expression levels of hENT-1 in PDAC samples (**A**) Representative pictures of PDAC epithelium and stroma before and after laser-assisted microdissection, performed on frozen tissue with the Leica LDM7000 microscope, as described previously [62]. (**B**) Comparison between hENT-1 gene expression values in microdissected (LMD) and non-microdissected (no LMD) samples from 22 PDAC tissues. Expression of hENT-1 was evaluated by Real-Time Quantitative PCR and normalized to GAPDH expression, as described previously [42]. *t*-test statistics was performed using Graph Pad version 7. Clinicopathological features of this subgroup of patients are reported in Table S1 [64,65,66]. (**C**) Comparison between hENT-1 protein expression values (evaluated by nanoLC-MS/MS) in LMD stromal and tumor compartments (* = *p* < 0.05) in *n* = 15 matched samples with respective survival curves. Protein values are represented in log10(LFQ). Patients were grouped in high and low hENT-1 protein expression according to the median cutoff. [62,67]. T-test statistics and Kaplan-Meier plots were performed in R version 3.5.2.

For instance, our PCR analysis of 22 non-microdissected (no LMD, including tumor and stroma tissues) samples showed a minor gene expression variability, with coefficient of variation values of the hENT-1 expression values ranging from 7% to 16% compared to the respective microdissected (LMD, including only tumor tissues) specimens (Figure 3A,B). This could potentially affect the stratification of the patients in the “low” vs. “high” expression categories and the correlation with clinical outcome. Additionally, proteomics analyses of LMD matched epithelial and stromal compartments showed an up-regulation (*p* = 0.017) of hENT-1 in the epithelial compartment (Figure 3C). Of note, although the presented cohort is relatively small (*n* = 13), epithelial hENT-1 expression was associated with significantly longer survival while no difference in the OS curve were observed for hENT-1 stromal expression (Figure 3C). Successful dissection of tumor and stromal compartment is reported in Figure S3. Recent studies showed the impact of LMD on the quality of both mRNA and protein content in PDAC specimens [61,62], and might explain why a not laser-assisted microdissection did not result in the association of hENT-1 expression levels with disease- specific survival, as reported by Jiraskova and collaborators in a retrospective study on a cohort of 69 resected PDAC patients treated with gemcitabine [63].

Of note, the data of this study also suggested a limited proportional dependence between hENT-1 gene expression evaluated by qRT-PCR in FFPE samples and protein levels as assessed by immunohistochemistry with the 10D7G2 antibody [63]. This is in agreement with the study by Raffenne and collaborators, where a higher degree of concordance was observed between hENT-1 mRNA expression levels and the SP120 rabbit clone [3]. These findings suggest that this antibody might recognize an unprocessed form of hENT-1 which is directly linked to the mRNA level, whereas the 10D7G2 clone recognizes the active glycosylated stabilized form, hence explaining its better predictive value. Remarkably, a significant correlation between mRNA level and IHC was also found, for another non-commercial rabbit anti-hENT-1 antibody developed by Pastor-Anglada and collaborators, who observed similar results in tumor cells with a different pathology [40,68].

Lastly, in the study by Raffenne and collaborators, the hENT-1 status was assessed by IHC using the 10D7G2 antibody also on coupled samples from both primary and metastatic tumors. Concordance between primary tumor and metastases was excellent for synchronous metastases. In contrast, a lower concordance was reported between metachronous metastases and primary tumors. As postulated by the authors, this discrepancy could be the result of the gemcitabine treatment that led to the selection of hENT-1-low clones in the metachronous metastases [3]. In this regard, further limitations might be represented by the relatively small sample size and other determinants influencing hENT-1 expression, such as disease stage and parameters discussed in Section 6. This is an extremely important aspect that could explain why the role of hENT-1 expression as a biomarker could not be validated when a comparison of gemcitabine with its lipophilic analog CO-101 was carried out within the prospective biomarker-stratified Low hENT1 Adenocarcinoma of the Pancreas (LEAP) trial, which enrolled PDAC patients in the metastatic setting [5]. However, another potential explanation of the lack of association between hENT-1 expression and response to gemcitabine is the use of the rabbit monoclonal antibody SP120, as reported above.

## 6. Factors Involved in hENT1 Regulation and Gemcitabine Activity

### 6.1. Genetics: Mutations and Polymorphisms

Structure and function studies have reported that hENT-1 transmembrane domains (TMDs 3–6) might be involved in interaction of nucleosides with the transporter [69]. Based on that, SenGupta et al. [70] explored the role of point mutations on glycine 179 and glycine 184 located in transmembrane domain five (TMD 5), using a GFP-tagged hENT-1 in a yeast nucleoside transporter assay system. As a result, substitution of glycine 179 with leucine, valine, or cysteine caused the lack of transporter activity without affecting its targeting to the plasma membrane. On the other hand, mutation of glycine 179 to alanine or serine influenced neither the activity of hENT-1 nor its targeting to plasma membrane. Hence, it could conceivably be suggested that glycine 179 may have an indirect but vital role in the permeation pathway of hENT-1.

Single nucleotide polymorphisms (SNPs) have been mentioned to affect the gene expression of hENT-1 and thus influencing gemcitabine clinical efficacy [71,72]. Three SNPs were confirmed in the proximal promoter of hENT-1 by Myers et al. [72]: −1345C > G, −1050G > A, and −706G > C. Higher expression levels were observed for two haplotypes (CGC, CAG) when cloning the four naturally occurring haplotypes (CGG, CAG, CGC, GAG) into a Luciferase expression system. Individuals with such haplotypes presented increased hENT-1 expression in comparison to those with normal haplotypes.

The distribution of variants in genes involved in gemcitabine pharmacology and their association with non-small lung cancer was further characterized by Soo et al. [73]. Their results revealed that the non-synonymous variant CNT1 + 1561 G > A is correlated with increased uptake of gemcitabine and hematologic toxicity. However, as the study was limited by the small sample size, larger studies are needed to validate these findings.

### 6.2. Epigenetics and microRNAs

#### 6.2.1. Epigenetics

The expression of drug transporters, drug metabolizing enzymes, and nuclear receptors, is under epigenetic control affecting the regulation of various genes and response to chemotherapeutic drugs [74]. The cellular levels of three histone modifications (H3K4me2, H3K9me2, H3K18ac), were examined by tissue microarrays from two cohorts with PDAC patients by Manuyakom and collaborators. Low H3K4me2 and H3K9me2 levels were associated with worse overall and disease-free survival (Adjusted HR: 1.48 and 1.44, respectively) [75]. Later, methylation of lysine H3K9 was extensively studied by Candelaria, et al. [76]. More specifically, they exposed CaLo cells to increasing concentrations of gemcitabine which eventually became resistant. This state was accompanied by down regulation of hENT-1. To determine whether gemcitabine resistance was associated with gene silencing induced by increased histone deacetylase activity, they performed ChIP assays, which finally showed a decrease in H3 and H4 acetylation at the hENT-1 promoter. They proposed that this mechanism could silence the expression of hENT-1 and therefore lead to gemcitabine resistance in cervical cancer cell lines.

#### 6.2.2. microRNAs

In the recent years it has become clear that protein expression levels can be regulated by microRNAs. microRNAs are small non-coding RNAs, of 19 to 25 nucleotides, that interact with the mRNA of coding genes, directing their post-translational repression. They are known to influence various cellular processes such as cell proliferation and cell death, mainly through negative regulation of gene expression [77]. In pancreatic cancer, several miRNAs have been reported to be aberrantly expressed, including miR-34 [78], miR-21, miR-155, miR-221, and miR-222 [79]. The regulation of nucleoside transporters by microRNAs is still poorly understood. Theoretically microRNAs could target the mRNA of nucleoside transporters, down-regulating their expression levels.

Using a collection of databases of microRNA-gene interactions (“multimir” R package), 175 miRNAs emerged as miRNA potentially targeting hENT-1 (Table S2). Of note, four of these microRNAs are overexpressed in PDAC as reported in Table 1.

Of note, the presence of tumor-derived microRNAs in both tissues and body fluids offers an opportunity for their potential application as liquid biopsy-based biomarkers, and future studies should evaluate whether emerging circulating microRNAs could be a useful tool for minimally-invasive estimation of hENT-1 levels and prediction of gemcitabine activity in PDAC patients.

MiR-196a-3p is up-regulated in exosomes of pancreatic cancer cell lines and in serum’s exosomes of localized PDAC patients (stage I and IIA, *n* = 15) when compared to healthy subjects (*n* = 15) [80]. However, data on outcome or to response to gemcitabine are missing.

Conversely, miR-23b-3p was found to correlate with pancreatic cancer progression in a cohort study in patients with chronic pancreatitis (CP) and pancreatic cancer. Furthermore, in this study, the authors did not provide data on gemcitabine activity, but assessed the expression level of miR-23b-3p in exosomes isolated from patients’ serum demonstrating the association of miR-23b-3p to CA19–9 levels [81].

High levels of miR-155–5p were associated to gemcitabine resistance in a study conducted by Mikamori and colleagues [82]. They reported three important findings: (i) long-term exposure to gemcitabine resulted in an increasing level of miR-155–5p; (ii) miR-155–5p levels were positively associated to exosome secretion that promoted gemcitabine resistance; (iii) increasing level of miR-155–5p in PDAC cell while blocking exosome secretion did not induce gemcitabine resistance. This later finding suggests an indirect activity which might be mediated by the miRNA mediated modulation of hENT-1 levels and deserves further research.

Similarly, Zhao and colleagues validated the antagomir for miR-221–5p (a group of miRNA antisense oligonucleotides) to restore chemosensitivity in gemcitabine-resistance cell lines. This miRNA was over-expressed in PDAC cancer-stem-cell subpopulation and regulated some stemness markers such as CDK6, C5ORF41, EFNA1, IRAK3, KLF12, MAPK10, NRP1, SMAD7, SOCS6 and ZBTB41 [83]. However, more research needs to be performed to determine the prognostic and/or predictive characteristics of both tissue and circulating microRNAs regarding their role in nucleoside transport.

### 6.3. Tumour Microenvironment

#### 6.3.1. Hypoxia

PDAC is characterized by a unique desmoplastic stroma and by the presence of an intense fibro-inflammatory reaction, known as desmoplastic reaction (DR). DR causes the continuous deposition of extracellular matrix components, including collagen type I and III, hyaluronic acid, and fibronectin, by activated pancreatic stellate cells (PSCs) [84]. As a result of increased intratumoral pressure and the subsequent compression of tumor vasculature, tumor cells experience hypoxia and metabolic stress [85]. Koong et al. were the first to observe hypoxia in PDAC [86], reporting areas of pancreatic carcinoma tissues with median pO_2_ levels of 0–5.3 mmHg. In contrast, normal tissues had a median pO_2_ level of 24 to 92.7 mmHg. Later, Buchler and collaborators [87] showed that hypoxia-inducible factor 1 (HIF-1), an important regulator of cellular response to hypoxia, is activated in PDAC in response to low oxygen conditions. High levels of HIF-1α promote angiogenesis via increased VEGF expression [87], hence promoting PDAC proliferation and metastatic potential [88]. Based on this rationale, clinical trials using drugs targeting angiogenesis have been conducted. However, despite promising preliminary results, anti-angiogenic drugs demonstrated low efficacy in PDAC. Low drug delivery due to vasculature collapse and poor tumor perfusion might explain, at least in part, the modest effectiveness of anti-angiogenic drugs (e.g., bevacizumab) in PDAC [89].

Remarkably, hypoxia also influences nucleoside transporters, as described by Eltzschig and collaborators [90]. Using in vitro and in vivo models of extracellular adenosine signalling, it was shown that hENT-1 and hENT-2 gene expression and function are negatively regulated by HIF-1α.

**Figure 4:**
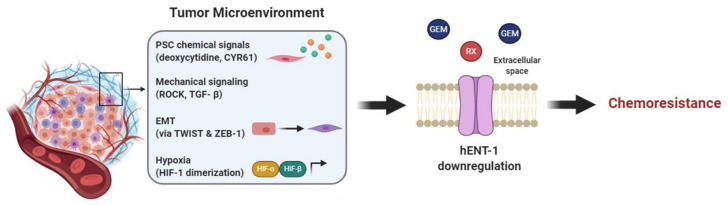
Tumor microenvironment affects hENT-1 expression causing chemoresistance. Components of the TME trigger PDAC hENT-1 downregulation and decreased activity. In order: PSCs secrete chemical factors, such as CYR61 and deoxycytidine, which downregulate hENT-1 and compete with drug metabolizing enzymes, respectively; mechanical signaling, such as TGF-β and ROCK, and EMT, triggered by TWIST and ZEB-1, also contribute to hENT-1 decreased activity; hypoxia induces HIF-α/HIF-B heterodimerization, which activates transcription of hypoxia-related genes further decreasing hENT-1 expression. Together these factors cause chemoresistance to drugs which are taken up via hENT-1 (e.g., gemcitabine (GEM) and RX-3117).

In particular, hENT-1 and hENT-2 are involved in the passage of adenosine through the endothelial membrane acting as bi-directional channels in normoxic conditions. In contrast, under hypoxic conditions the adenosine movement is unidirectional but predominantly inward because the extracellular adenosine concentration is much higher than the intracellular. Therefore, the repression of NTs induced by hypoxia causes an extracellular increase of adenosine concentration and signaling effects. Additionally, HIF-α forms a heterodimer with HIF-β during hypoxia which also causes the nuclear translocation of HIF-1 and the binding to the promoter of hypoxia-responsive element of hENT-1 (Figure 4). Thus, a downregulation of hENT1 occurs and consequently a decrease of adenosine uptake. These mechanisms represent a transcriptional pathway to limit the inflammatory response and to ensure the integrity of the vascular barrier during hypoxic conditions [91].

#### 6.3.2. Mechanobiology

PDAC stroma, which accounts for the majority of tumor mass, is involved in resistance to gemcitabine, and a recent publication by Dalin et al. suggested a role of PSCs through an indirect influence on hENT-1. In this case the stroma compartment does not directly affect hENT-1 expression, but most likely bypasses it by producing elevated amounts of deoxycytidine. High deoxycytidine levels are taken up by PDAC cells, via hENT-1, and compete with gemcitabine’s intracellular pool for phosphorylation and activation of the drug, therefore causing resistance [92]. Although further investigations are necessary [93] these promising preliminary results suggest that hENT-1 activity is indirectly influenced by the tumor stroma and that this interaction has a relevant role for PDAC gemcitabine chemoresistance.

Mechanical cues and hENT-1 are also intertwined, playing an additional role in PDAC EMT and resistance to gemcitabine. EMT is characterized by cellular physical changes, e.g., viscoelastic and stiffness properties modulation [94]. Notably, hENT-1 has been reported as a regulator of cellular mechanical properties, by means of EMT induction. Indeed, knockdown of hENT-1 was shown to induce cell elongation, stiffness reduction, increased migration potential and expression of EMT markers [95]. Consistently, recent studies reported that gemcitabine resistant cells, which had hENT-1 downregulation, were accompanied by an EMT phenotype. Additionally, these studies highlighted that the EMT was triggered by TWIST and ZEB-1 transcription factors. By inhibiting TWIST and ZEB-1 the cells had an increased hENT-1 expression and reversed EMT phenotype, increasing gemcitabine efficacy [96,97]. These interesting results suggest that reversing cellular mechanical changes, i.e., EMT, could at least in part contrast the phenomenon of gemcitabine resistance relying on hENT-1.

Lastly, mechanical signaling, that is signaling induced by or causing mechanical stress is also involved in hENT-1 regulation. In lung cancer, hENT-1 together with ROCK1-Rho A—kinases involved in actin organization, cell contractility and motility—are regulated by miR-26b. miR-26b mimic indeed is responsible for downregulation of the aforementioned proteins, leading to reduced tumor invasion and migration [98]. The exact mechanism behind hENT-1-ROCK interaction and whether this is also valid in PDAC has yet to be confirmed. Nevertheless, since both hENT-1 and ROCK are generally overexpressed in PDAC it is not a surprise that they are involved in the aggressive behavior and chemoresistance of this tumor type. Moreover, experimental data showed that in PDAC at least one mechanical signaling pathway is responsible for hENT-1 regulation. PSCs in the tumor microenvironment are a source of cysteine-rich angiogenic inducer 61 (CYR61) and this is due to upregulated TGF-β signaling, a pathway involved in mechanical signaling, ECM remodeling and motility. Hesler et al. reported that this high concentration of CYR61 affected PDAC cells by downregulating hENT-1, therefore causing gemcitabine chemoresistance [99].

In conclusion, emerging evidence highlight the interaction of hENT-1 with tumor stroma and mechanical signaling, yet more evidence has to be obtained to have potential new targets to overcome pancreatic cancer progression and chemoresistance (Figure 4).

## 7. Discussion

The increasing use of a non-gemcitabine-based therapeutic option (FOLFIRINOX), both in the palliative and adjuvant setting, should prompt further development of predictive biomarkers for gemcitabine-based regimens. The validation of these biomarkers may indeed pave the road to the selection of patients who are likely to receive a benefit from gemcitabine-based adjuvant chemotherapy, and therefore has an immediate clinical relevance.

Remarkably, results obtained in several studies demonstrate that hENT-1 expression level is predictive of gemcitabine benefit, but only when assessed with the 10D7G2 mouse clone. Nevertheless, since this clone is not commercially available, this approach is not clinically-feasible and other strategies for the evaluation of hENT-1 status should be investigated. The quantification of hENT-1 mRNA levels could represent an alternative technique to IHC for the clinical evaluation of hENT-1 status in PDAC patients. A predictive role for hENT-1 mRNA expression in the treatment of PDAC with gemcitabine was previously reported in PDAC laser-microdissected specimens [42]. However, Raffenne and collaborators found that the threshold required to achieve statistical significance was higher with qRT-PCR compared with IHC, probably because no laser-microdissection was performed, leading to heavy microenvironment contamination and falsely decreased mRNA level [3]. In this regard, future studies involving laser microdissection techniques and larger cohorts of patients are required to validate the analysis of mRNA as a potential strategy for the clinical assessment of hENT-1 status.

Several additional factors are involved in cancer cell sensitivity to gemcitabine. For instance, the expression level and activity of gemcitabine-activating enzymes such as deoxycytidine kinase (dCK), and inactivating enzymes such as cytidine deaminase (CDA) and nucleotidase (NT5C1A/NT5C3) may provide a rational explanation for the discrepancy between high hENT-1 level and the poor response to gemcitabine-based chemotherapies [46]. In this regard, the study of more complex gemcitabine sensitivity/resistant signatures using both preclinical models and clinical samples, as well as novel technologies is warranted. For instance, a recent study on proteomics in gemcitabine resistant PANC1 cells and xenografts did not show a significant increase (*p* = 0.29) of hENT-1 expression in the resistant cells, though the fold-change was 1.5, while revealing that proteins associated with gemcitabine resistance are correlated with microtubule regulation [100]. Notably, this data provides an explanation as to why the combination of gemcitabine with nab-paclitaxel is effective in PDAC patients. However, hENT-1 is trafficked to the plasma membrane in association with microtubules suggesting a potential correlation with this system [101]. These findings are extremely interesting because another main challenge for the use of hENT-1 as a clinical biomarker is its validation in patients undergoing treatments with combination of gemcitabine and nab-paclitaxel and not only with gemcitabine monotherapy.

The role played by different cancer cell subpopulations, such as CSCs, as well as non-cancerous cells within the TME, in gemcitabine efficacy represents another critical issue, requiring further elucidation. Of note, recent studies showed that extracellular vesicles (EVs) can confer resistance to gemcitabine by miR-155-mediated suppression of dCK, which catalyzes the rate-limiting reaction in gemcitabine activation [102]. Future studies should investigate whether miRNAs affecting hENT-1 expression could also be involved in the transfer of a resistant phenotype through EVs.

The reliability of a predictive biomarker is assessed through the analysis of sensitivity, specificity, and positive and negative predictive values. However, these parameters need extensive validation studies and quality assessment prior to approval and application in clinical setting. Therefore, the expression levels of hENT-1 should be evaluated within trials testing therapeutic strategies/drugs than can bypass hENT-1 mediated gemcitabine resistance. For instance, NUC-1031 (Acelarin^®^, NuCana, Edinburgh, UK), which is the first anti-cancer ProTide to enter the clinic, is a phosphoramidate modification of gemcitabine designed to overcome several mechanisms affecting gemcitabine efficacy. According to pre-clinical data, the increased hydrophobicity of NUC-1031 allows it to enter the cells bypassing the hENT-1 transporter [6], similarly to the previously tested lipid-drug conjugate CO-101 (CP-4126) [103,104]. However, the trials on CO-101 resulted in a disappointing failure in PDAC patients. In particular, in a randomized prospective study in patients with untreated metastatic PDAC, CO-101 was not superior to gemcitabine in the low tumor hENT-1 (assessed with the SP120 antibody) and an adverse effect profile similar to gemcitabine [5] was found, such as recently described for NUC-1031. Of note, hENT-1 is not the only potential mechanism mediating the favorable effect of NUC-1031, since this drug does not require the phosphorylation to difluorodeoxycytidine monophosphate (dFdCMP) by dCK and it preserves higher concentrations of the active triphosphate metabolite (dFdCTP) than gemcitabine at equimolar doses inside the tumor cells [6,105]. However, in the future trials on this drug in PDAC patients, standardized IHC technique for the detection of hENT-1 would be essential to overcome the criticisms about the previous CO-101 trial.

Several studies support the association between no or low hENT-1 expression in tumors and poor response to gemcitabine in other cancers, including bladder, biliary tract, and lung cancers. Thus, the validation of hENT-1 standardized IHC techniques would be useful also for other cancer types. For instance, Matsumura and colleagues evaluated the predictive potential of hENT-1 expression in patients with metastatic bladder cancer treated with gemcitabine-cisplatin-based combination chemotherapy. The IHC results showed that hENT-1 was localized in the cytoplasm of bladder tumor cells, and patients with high hENT-1 expression levels had a significantly longer median survival (17.3 months) compared to patients with lower levels (11.6 months) [106]. Similar results were observed by the IHC analysis of hENT-1 in a panel of patients with advanced Biliary Tract Cancer (BTC). Moreover, this study suggested that hENT-1 mediates the intracellular transport not only of gemcitabine but also of capecitabine, because a subpopulation of BTC patients treated with these two drugs showed a correlation between hENT-1 and OS [107]. Finally, Oguri and colleagues evaluated the hENT-1 expression in non-small cell lung cancer (NSCLC) patients who received gemcitabine-containing chemotherapy, showing that the absence of hENT-1 expression may be useful to predict resistance to gemcitabine-containing chemotherapy in NSCLC [108]. Of note this study showed that the protein expression of hENT-1 in a panel of cell lines with acquired resistance to different drugs, including gemcitabine, cisplatin and paclitaxel, was similar to the expression levels in their respective parental cells. These results suggest that hENT-1 is important in inherent resistance, but does not have a role in acquired resistance. Moreover, this transporter might still be used as potential predictive biomarker of gemcitabine efficacy also after pretreatment with other drugs, such as after neoadjuvant regimens, which are gaining momentum in the multidisciplinary treatment of even potentially resectable PDAC [109].

## 8. Conclusions

In the present article, we explored the contradictory data related to the predictive value of hENT-1 for gemcitabine activity in PDAC. We also considered the issues related to commercial and not commercial antibodies for IHC and laser-microdissected specimens for PCR analysis. Finally, we discussed the potential impact of different biological mechanisms on hENT-1 regulation, supporting the need of integrating additional (tissue and circulating) biomarkers and further exploring the uncertainty regarding the clinical significance within larger prospective trials using standardized methodologies.

The emergence of “omics” technologies (i.e., genomics, transcriptomics, proteomics, and metabolomics) has encouraged the discovery of new biomarkers. However, the identification of solid and reproducible molecular markers is amongst the biggest challenges in personalized cancer medicine. Therefore, in the present article, we have also reported molecular mechanisms influencing the expression and activity of hENT-1, because we reckon that the integration of existing molecular knowledge should help to adjust for clinical data heterogeneity and limitation.

Last but not least, as reported in the tale of Ali Baba, in order to discover the secret of the cave, he was at right place at right time, suggesting that a relentless pursuit of the goals will lead to achieving success. Thus, persistent and appropriate studies are needed in order to validate effective biomarkers and will hopefully guide the selection of the best (sequence of) anticancer therapy in PDAC patients.

## Figures and Tables

**Table 1:** MicroRNAs potentially influencing hENT1 expression.

microRNA	microRNA acc	Experiment Type	Database Source	Comments	Reference
hsa-miR-196a-3p	MIMAT0004562	PAR-CLIP	mirtarbase	Up-regulated in exosomes of PDAC’s serum	[67]
hsa-miR-221-5p	MIMAT0004568	Degradome sequencing	tarbase	Up-regulated in PDAC cancer stem cells	[70]
hsa-miR-23b-3p	MIMAT0000418	Degradome sequencing	tarbase	Up-regulated in exosomes of PDAC’s serum and correlated to CA19–9	[68]
hsa-miR-155-5p	MIMAT0000646	Degradome sequencing	tarbase	Up-regulated in GEM resistant PDAC cells	[69]

## Supplementary Materials

The following are available online at https://www.mdpi.com/2072-6694/12/11/3206/s1, Table S1: Clinicopathological characteristics of PDAC patients evaluated for hENT-1 mRNA levels. Table S2: List of MicroRNA Targeting hENT-1. Figure S1: Chemical structures of the drugs transported by nucleoside transporters. Figure S2: Studies evaluating hENT-1 expression levels in pancreatic tumors and normal specimens. Figure S3: Stromal and tumor makers for accurate dissection.
