# Supplementary material for: “Open Sesame?”: Biomarker Status of the Human Equilibrative Nucleoside Transporter-1 and Molecular Mechanisms Influencing its Expression and Activity in the Uptake and Cytotoxicity of Gemcitabine in Pancreatic Cancer
Source: Cancers (Basel). 2020 Oct 31;12(11):3206. doi: 10.3390/cancers12113206 (PMC7692081; doi:10.3390/cancers12113206)
Supplement: Supplementary file 1 [file cancers-12-03206-s001.zip › cancers-965135-Supplementary/cancers-965135-Supplementary.docx]

Supplementary Materials: “Open Sesame?”: Biomarker Status of Influencing the Human Equilibrative Nucleoside Transporter-1 and Molecular Mechanisms Influencing its Expression and Activity in the Uptake and Cytotoxicity of Gemcitabine in Pancreatic Cancer

Ornella Randazzo, Filippo Papini, Giulia Mantini, Alessandro Gregori, Barbara Parrino, Daniel S.K. Liu, Stella Cascioferro, Daniela Carbone, Godefridus J. Peters, Adam E. Frampton, Ingrid Garajova and Elisa Giovannetti


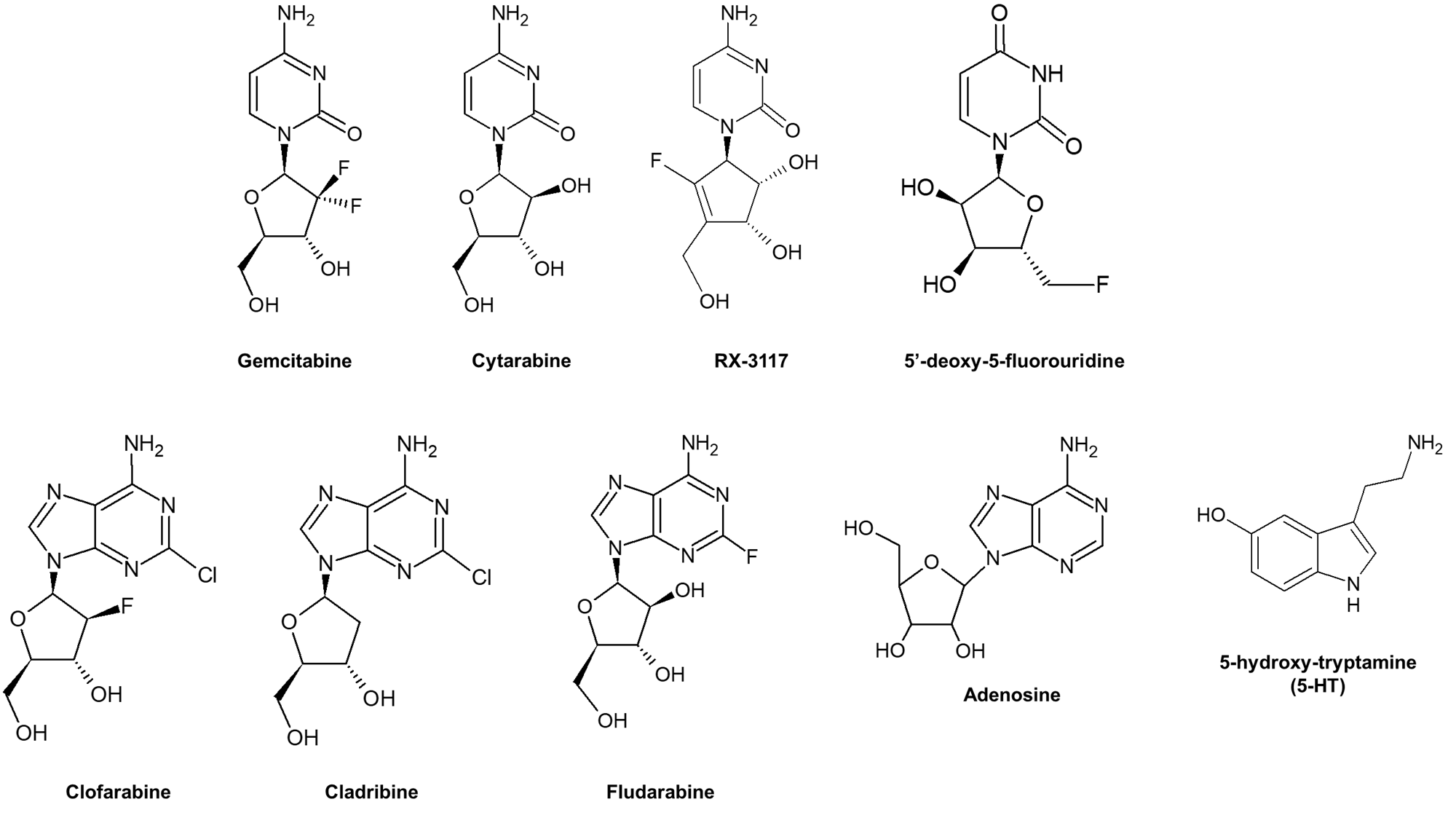


**Figure S1.** Chemical structures of the drugs transported by nucleoside transporters**.**


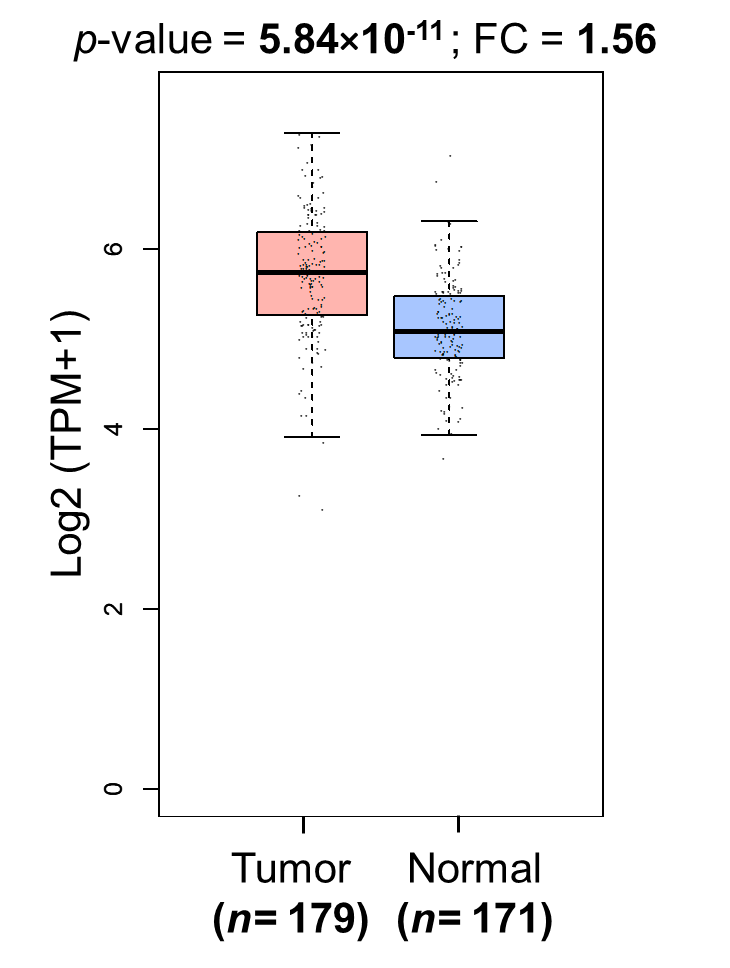


**B**

**A**

**Figure S2.** Studies evaluating hENT-1 expression levels in pancreatic tumors and normal specimens. The nucleoside transporter hENT-1iis overexpressed in different tumor types, including PDAC analyzing the RNA sequencing expression data of 9,736 tumors and 8,587 normal samples from the TCGA and the GTEx projects (http://gepia.cancer-pku.cn/detail.php?gene=SLC29A1) (panel A). However, the analysis of similarly matched transcriptomics and proteomics public datasets did not show a significance difference in hENT-1 expression levels (panel B).


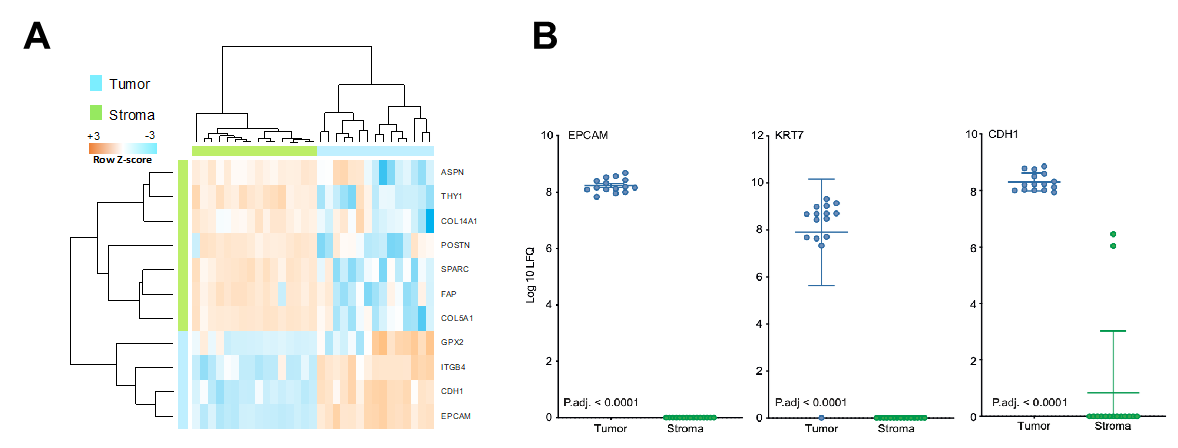


**Figure S3.** Stromal and tumor compartment dissection was confirmed by evaluating the protein expression of epithelial and stromal markers (panel **A**). Moreover, protein expression of specific tumor markers (EPCAM, KRT7 and CDH1) in matched stromal samples ranged from zero to very low levels, indicating a minimal stromal contamination (panel **B**).

**Table S1.** Clinicopathological Characteristics of PDAC Patients Evaluated for hENT-1 mRNA Levels. The expression of hENT-1 was evaluated by quantitative PCR, as described previously [1].

| **Age—years** |  |
| --- | --- |
| Mean (± SD) | 65 (± 5) |
| **Sex—No. (%)** |  |
| Male | 12 (54.5) |
| Female | 10 (45.5) |
| **Stage *—No. (%)** |  |
| II | 11 (50) |
| III | 11 (50) |
| **Grading (%)** |  |
| G1-G2§ | 9 (40.9) |
| G3 | 13 (59.1) |

Notes: *AJCC Cancer Staging Manual, 7^th^ Edition; §WHO grading system 2007; Abbreviations: PDAC = pancreatic ductal adenocarcinoma, No. = number of patients.

Table S2 can be found in excel.

Reference

**1**. Toffalorio, F.; Giovannetti, E.; De Pas, T.; Radice, D.; Pelosi, G.; Manzotti, M.; Minocci, D.; Spaggiari, L.; Spitaleri, G.; Noberasco, C.; et al. Expression of gemcitabine- and cisplatin-related genes in non-small-cell lung cancer. *Pharmacogenomics J.* **2010**, *10*, 180–190, doi: 10.1038/tpj.2009.53.
